# Effect of tumor size on breast cancer-specific survival stratified by joint hormone receptor status in a SEER population-based study

**DOI:** 10.18632/oncotarget.3945

**Published:** 2015-05-11

**Authors:** Yi-Zi Zheng, Lei Wang, Xin Hu, Zhi-Ming Shao

**Affiliations:** ^1^ Department of Breast Surgery, Key Laboratory of Breast Cancer in Shanghai, Fudan University Shanghai Cancer Center, Fudan University, Shanghai, China; ^2^ Department of Oncology, Shanghai Medical College, Fudan University, Shanghai, China; ^3^ Institutes of Biomedical Science, Fudan University, Shanghai, China

**Keywords:** breast cancer, hormone receptor status, tumor size, breast cancer-specific mortality

## Abstract

**Background & Aims:**

The prognostic value of tumor size is variable. We aimed to characterize the interaction between tumor size and hormone receptor (HoR) status to determine breast cancer-specific mortality (BCSM).

**Methods:**

We used the Surveillance, Epidemiology and End Results (SEER) registry to identify 328, 870 female patients diagnosed with invasive breast cancer from 1990 through 2010. Primary study variables included tumor size, joint HoR status and their corresponding relationship. Kaplan-Meier and adjusted Cox proportional hazards models with interaction terms were utilized.

**Results:**

The multivariable analysis revealed a significant interaction between tumor size and HoR status (*P* < 0.001). Using tumors 61–70 mm in size as the reference for estrogen receptor-negative (ER−) and progesterone receptor-negative (PR−) disease, the hazard ratio (HR) for BCSM increased with increasing tumor size across nearly all categories. In the ER-positive (ER+) and PR-positive (PR+) group, however, patients with tumors > 50 mm had nearly identical BCSM rates (*P* = 0.127, *P* = 0.099 and *P* = 0.370 for 51–60 mm, 71–80 mm and > 80 mm tumors, respectively), whereas BCSM was positively correlated with tumors < 51 mm.

**Conclusions:**

The observation of identical HRs for BCSM among patients with ER+ and PR+ tumors >50 mm underscores the importance of individualized treatment. Our findings may contribute to a better understanding of breast cancer biology.

## INTRODUCTION

To date, numerous breast cancer prognostic factors have been identified, including tumor size, degree of axillary lymph node (LN) involvement, age, histologic grade, hormone receptor (HoR) status, HER2/neu status, and the presence of lymphovascular invasion [1]. However, it is difficult to predict metastasis and outcomes in this heterogeneous disease. Given the urgent need for individually tailored therapy, cancer-specific outcomes must be estimated more accurately.

Traditionally, tumor size has served as one of the most powerful prognostic factors in breast cancer [2, 3, 4]; accordingly, this factor serves as the basis of major staging systems [5, 6, 7]. Increasing tumor size has been reported to be associated with increased breast cancer-specific mortality (BCSM) within each joint estrogen receptor (ER) and progesterone receptor (PR) status category [8, 9]. However, these data are from studies in which the investigators placed all stage T3 tumors (>5 cm) in one category [8, 9]. The prognostic value of tumor size is currently being reconsidered. In particular, the view that breast tumor size correlates with survival in all subtypes of breast cancer has been questioned [10]. Indeed, several studies have noted that this pattern does not hold for small breast tumors [11, 12, 13] and that luminal breast cancer is a highly heterogeneous disease [14]. The fact that luminal tumors are ER and/or PR positive prompted us to estimate the impact of HoR status on the prognostic value of large tumor size by another means. As limited study population sizes and recruitment periods have impeded subgroup analyses, we utilized Surveillance, Epidemiology, and End Results (SEER) population-based data to further clarify the impact of tumor size and HoR status on breast cancer prognosis. Therefore, we developed a more complete understanding of the impact of tumor size on survival over a wider size range. ER and PR are correlated in breast cancer and converge on common pathways, and there is increasing awareness that progesterone is an important hormone in breast cancer [15, 16]. Accordingly, HoR status was analyzed as the joint ER and PR status in our study.

## RESULTS

### Clinicopathologic patient parameters

In total, 328, 870 eligible female patients with invasive breast cancer were enrolled; 36, 509 of these patients died of breast cancer. The median follow-up period was 68 months. HoR status was analyzed based on joint ER and PR statuses (ER+PR+, ER+PR−, ER−PR+, and ER−PR−). Patient demographics and pathologic features based on ER/PR phenotypes are summarized in Table 1.

**Table 1:** Demographic and tumor characteristics of the study cohort

Characteristics	Number of patients (%)					*P*-value^a^
	Total	ER+PR+	ER+PR−	ER−PR+	ER−PR−	
	(*N* = 328870)	(*N* = 209883)	(*N* = 39336)	(*N* = 6252)	(*N* = 73399)	
Year of diagnosis						< 0.001
1990–1995	38666 (11.8)	23991 (11.4)	4922 (12.5)	1580 (25.3)	8173 (11.1)	
1996–2000	55713 (16.9)	35389 (16.9)	6501 (16.5)	1424 (22.8)	12399 (16.9)	
2001–2005	102695 (31.2)	63788 (30.4)	12589 (32.0)	1736 (27.8)	24582 (33.5)	
2006–2010	131796 (40.1)	86715 (41.3)	15324 (39.0)	1512 (24.2)	28245 (38.5)	
Race						< 0.001
White	269184 (81.9)	176257 (84.0)	32330 (82.2)	4822 (77.1)	55775 (76.0)	
Black	30898 (9.4)	14797 (7.1)	3772 (9.6)	814 (13.0)	11515 (15.7)	
Other^b^	27362 (8.3)	17845 (8.5)	3085 (7.8)	595 (9.5)	5837 (8.0)	
Unknown	1426 (0.4)	984 (0.5)	149 (0.4)	21 (0.3)	272 (0.4)	
Marital status						< 0.001
Married	190628 (58.0)	121717 (58.0)	21928 (55.7)	3839 (61.4)	43144 (58.8)	
Not married^c^	128172 (39.0)	81633 (38.9)	16271 (41.4)	2260 (36.1)	28008 (38.2)	
Unknown	10070 (3.1)	6533 (3.1)	1137 (2.9)	153 (2.4)	2247 (3.1)	
Age						< 0.001
< 20	2078 (0.6)	988 (0.5)	209 (0.5)	72 (1.2)	809 (1.1)	
30–39	20890 (6.4)	10925 (5.2)	1952 (5.0)	762 (12.2)	7251 (9.9)	
40–49	68460 (20.8)	43766 (20.9)	5593 (14.2)	1908 (30.5)	17193 (23.4)	
50–59	83461 (25.4)	50672 (24.1)	10384 (26.4)	1580 (25.3)	20825 (28.4)	
60–69	71716 (21.8)	47215 (22.5)	9562 (24.3)	993 (15.9)	13946 (19.0)	
70–79	53309 (16.2)	36499 (17.4)	7328 (18.6)	604 (9.7)	8878 (12.1)	
>80	28956 (8.8)	19818 (9.4)	4308 (11.0)	333 (5.3)	4497 (6.1)	
Laterality						< 0.001
Left	167111 (50.81)	105943 (50.5)	20013 (50.9)	3276 (52.4)	37879 (51.6)	
Right	161721 (49.17)	103913 (49.5)	19322 (49.1)	2976 (47.6)	35510 (48.4)	
Only one side NOS	38 (0.00011555)	27 (0.0)	1 (0.0)	0 (0.0)	10 (0.0)	
Grade						< 0.001
I	55743 (16.9)	47729 (22.7)	6123 (15.6)	432 (6.9)	1459 (2.0)	
II	129182 (39.3)	98963 (47.2)	15785 (40.1)	1666 (26.6)	12768 (17.4)	
III and UD	129279 (39.3)	53997 (25.7)	15566 (39.6)	3747 (59.9)	55969 (76.3)	
Unknown	14666 (4.5)	9194 (4.4)	1862 (4.7)	407 (6.5)	3203 (4.4)	
Tumor size (mm)						< 0.001
0–10	84296 (25.6)	59931 (28.6)	10552 (26.8)	1281 (20.5)	12532 (17.1)	
11–20	127576 (38.8)	86909 (41.4)	14452 (36.7)	2300 (36.8)	23915 (32.6)	
21–30	65348 (19.9)	37914 (18.1)	7755 (19.7)	1408 (22.5)	18271 (24.9)	
31–40	24587 (7.5)	12487 (5.9)	3059 (7.8)	567 (9.1)	8474 (11.5)	
41–50	11410 (3.5)	5567 (2.7)	1465 (3.7)	273 (4.4)	4105 (5.6)	
51–60	6507 (2.0)	3019 (1.4)	835 (2.1)	168 (2.7)	2485 (3.4)	
61–70	3316 (1.0)	1537 (0.7)	416 (1.1)	94 (1.5)	1269 (1.7)	
71–80	2335 (0.7)	1075 (0.5)	297 (0.8)	73 (1.2)	890 (1.2)	
>80	3495 (1.1)	1444 (0.7)	505 (1.3)	88 (1.4)	1458 (2.0)	
Regional nodes						< 0.001
Negative	213746 (65.0)	139868 (66.6)	25294 (64.3)	3794 (60.7)	44790 (61.0)	
Positive	109817 (33.4)	66427 (31.6)	13347 (33.9)	2347 (37.5)	27696 (37.7)	
Unknown	5307 (1.6)	3588 (1.7)	695 (1.8)	111 (1.8)	913 (1.2)	
Radiation						< 0.001
Yes	172895 (52.6)	113858 (54.2)	19986 (50.8)	3026 (48.4)	36025 (49.1)	
No	146850 (44.7)	90665 (43.2)	18265 (46.4)	3053 (48.8)	34867 (47.5)	
Unknown	9125 (2.8)	5360 (2.6)	1085 (2.8)	173 (2.8)	2507 (3.4)	

Abbreviations: ER, estrogen receptor; PR, progesterone receptor, UD, undifferentiated

a *P* value of the χ2 test comparing the ER+PR+, ER+PR−, ER−PR+ and ER−PR− groups

b Including American Indian/Alaskan native, and Asian/Pacific Islander

c Including divorced, separated, single (never married), and widowed.

### Clinicopathologic differences between groups

As illustrated in Table 1, 63.82% (*n* = 209, 883) of the patients were ER+PR+, 11.96% (*n* = 39, 336) were ER+PR−, 1.9% (*n* = 6, 252) were ER−PR+, and 22.32% (*n* = 73, 399) were ER−PR−. There were statistically significant differences in all the variables across the four groups (*P* < 0.001). Compared with the ER−PR− group, the ER+PR+ group had smaller tumors (more tumors ≤2 cm in size: 70% vs 49.7%), less advanced disease (more grade I and II: 69.9% vs 19.4%) and less lymph node involvement (fewer positive nodes: 31.6% vs 37.7%).

### Impact of tumor size on breast cancer survival outcomes

**Figure 1:**
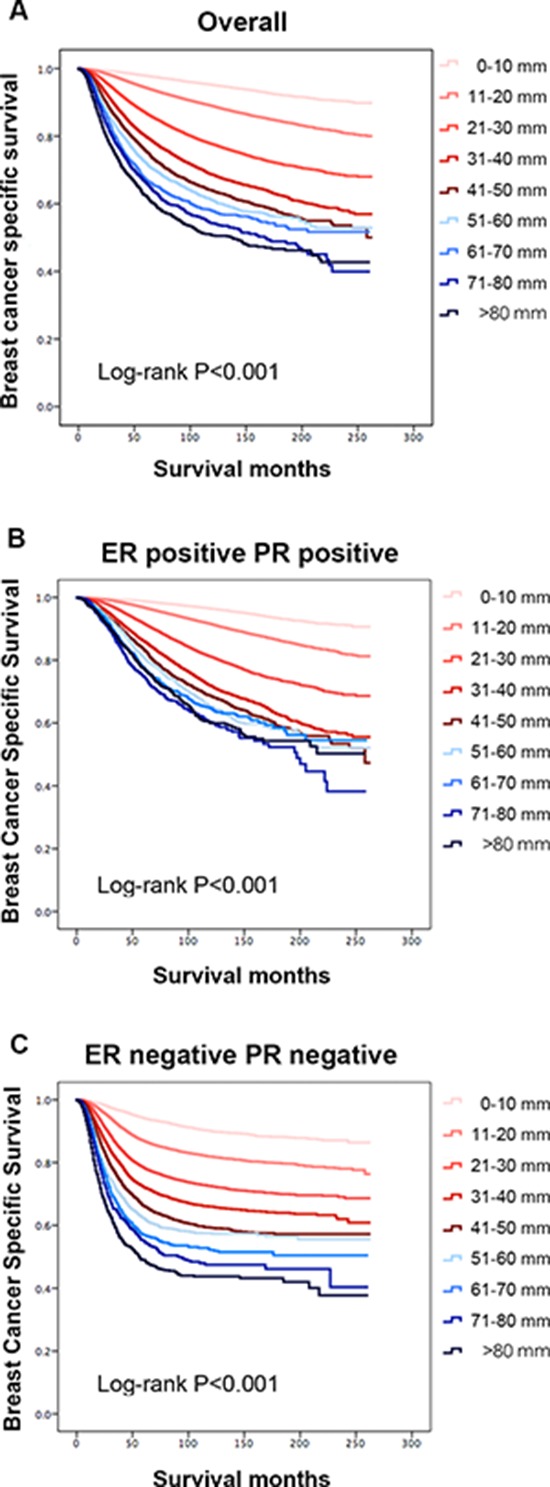
Patient survival curves according to tumor size **A.** The entire cohort, *P* < 0.001. **B.** The estrogen receptor (ER)-positive and progesterone receptor (PR)-positive group, *P* < 0.001. **C.** The ER-negative and PR-negative group, *P* < 0.001.

Kaplan-Meier analysis was used to determine breast cancer-specific survival (BCSS) in the groups based on tumor size (Figure 1A). Individual survival curves for the four ER/PR joint subgroups were generated (Figure 1B–C; Figure S1A–B). As expected, patients with 0 to 10 mm tumors exhibited the best survival rates (Figure 1A), while those with tumors greater than 80 mm exhibited the worst survival rates in the entire study cohort (*P* < 0.001). Unexpectedly, the stratified analysis indicated that patients with ER+PR+ tumors in the 50 to 80 mm groups experienced similar survival rates, whereas ER−PR− patients experienced increased breast cancer-specific mortality (BCSM) as tumor size increased throughout all size categories (*P* < 0.001).

We used the 61 to 70 mm group as the reference for univariate and multivariate analyses based on the Kaplan-Meier results. In the univariate analysis, the year of diagnosis, race, marital status, age, laterality, tumor size, tumor grade, ER and PR statuses, LN status and history of radiation were significantly associated with BCSS (*P* < 0.001). A multivariate analysis was performed using the Cox regression model. All the factors mentioned above were identified as independent prognostic factors (Table 2), including year of diagnosis (1996–2000, hazard ratio (HR) 0.760, 95% confidence interval (CI) 0.737–0.783; 2001–2005, HR 0.625, 95% CI 0.607–0.643; 2006–2010, HR 0.514, 95% CI 0.495–0.533), race (African-American, HR 1.296, 95% CI 1.258–1.336; others, HR 0.852, 95% CI 0.818–0.888), marital status (not married, HR 1.143, 95% CI 1.118–1.168), age (30–39 years, HR 0.893, 95% CI 0.806–0.988; 40–49 years, HR 0.770, 95% CI 0.698–0.850; 50–59 years, HR 0.827, 95% CI 0.750–0.913; 60–69 years, HR 0.968, 95% CI 0.877–1.069; 70–79 years, HR 1.255, 95% CI 1.136–1.387; > 80 years, HR 1.847, 95% CI 1.668–2.045), laterality (right, HR 0.971, 95% CI 0.951–0.991), grade (II, HR 1.960, 95% CI 1.853–2.074; III and undifferentiated, HR 2.928, 95% CI 2.767–3.098), HoR status (ER+PR−, HR 1.554, 95% CI 1.280–1.887; ER−PR+, HR 1.687, 95% CI 1.175–2.423; ER−PR−, HR 1.982, 95% CI 1.729–2.273), tumor size (0–10 mm, HR 0.185, 95% CI 0.164–0.208; 11–20 mm, HR 0.324, 95% CI 0.290–0.361; 21–30 mm, HR 0.545, 95% CI 0.489–0.607; 31–40 mm, HR 0.755, 95% CI 0.675–0.845; 41–50 mm, HR 0.845, 95% CI 0.750–0.953; 51–60 mm, HR 0.911, 95% CI 0.801–1.038; 71–80 mm, HR 1.160, 95% CI 0.994–1.354; > 80 mm, HR 1.079, 95% CI 0.931–1.249), LN involvement (positive, HR 2.478, 95% CI 2.422–2.536) and history of radiation (no radiation, HR 1.176, 95% CI 1.150–1.201). In the univariate analysis, a straightforward dose-effect relationship was observed between larger tumor size and increasing BCSM; however, the HR observed in the multivariate analysis was piecewise. For tumors less than 51 mm in size, the HR for BCSM increased with size from 0.185 (*P* < 0.001) in the 0–10 mm group to 0.845 (*P* = 0.006) in the 41–50 mm group. Thereafter, the HRs were not significantly different in the 51–60 mm group (HR 0.911, 95% CI 0.801–1.038, *P* = 0.161), the 71–80 mm group (HR 1.160, 95% CI 0.994–1.354, *P* = 0.059) or the > 80 mm group (HR 1.079, 95% CI 0.931–1.249, *P* = 0.313). These results were essentially consistent with the aforementioned Kaplan-Meier analysis.

**Table 2:** Cox proportional hazards regression model analysis of breast cancer-specific mortality

Variables	Univariate analysis		Multivariate analysis	
	HR (95% CI)	*P*-value	HR (95% CI)	*P*-value
Hormone receptor status				
ER+PR+	Reference	–	Reference	–
ER+PR−	1.663 (1.611–1.717)	< 0.001	1.554 (1.280–1.887)	< 0.001
ER−PR+	2.151 (2.028–2.280)	< 0.001	1.687 (1.175–2.423)	0.005
ER−PR−	2.694 (2.634–2.756)	< 0.001	1.982 (1.729–2.273)	< 0.001
Year of diagnosis				
1990–1995	Reference	–	Reference	–
1996–2000	0.701 (0.681–0.721)	< 0.001	0.760 (0.737–0.783)	< 0.001
2001–2005	0.593 (0.577–0.610)	< 0.001	0.625 (0.607–0.643)	< 0.001
2006–2010	0.472 (0.456–0.489)	< 0.001	0.514 (0.495–0.533)	< 0.001
Race				
White	Reference	–	Reference	–
Black	1.820 (1.767–1.874)	< 0.001	1.296 (1.258–1.336)	< 0.001
Other^a^	0.860 (0.826–0.896)	< 0.001	0.852 (0.818–0.888)	< 0.001
Unknown	0.338 (0.253–0.453)	< 0.001	0.361 (0.270–0.484)	< 0.001
Marital status				
Married	Reference	–	Reference	–
Not married^b^	1.379 (1.351–1.409)	< 0.001	1.143 (1.118–1.168)	< 0.001
Unknown	1.194 (1.121–1.271)	< 0.001	1.107 (1.039–1.179)	0.002
Age				
< 20	Reference	–	Reference	–
30–39	0.766 (0.692–0.848)	< 0.001	0.893 (0.806–0.988)	0.028
40–49	0.497 (0.450–0.548)	< 0.001	0.770 (0.698–0.850)	< 0.001
50–59	0.453 (0.411–0.500)	< 0.001	0.827 (0.750–0.913)	< 0.001
60–69	0.448 (0.406–0.494)	< 0.001	0.968 (0.877–1.069)	0.518
70–79	0.545 (0.493–0.601)	< 0.001	1.255 (1.136–1.387)	< 0.001
>80	0.904 (0.817–0.999)	0.048	1.847 (1.668–2.045)	< 0.001
Laterality				
Left	Reference	–	Reference	–
Right	0.959 (0.940–0.979)	< 0.001	0.971 (0.951–0.991)	0.005
Only one side, NOS	0.853 (0.275–2.644)	0.782	1.011 (0.326–3.137)	0.984
Grade				
I	Reference	–	Reference	–
II	3.074 (2.907–3.251)	< 0.001	1.960 (1.853–2.074)	< 0.001
III and UD	7.277 (6.895–7.680)	< 0.001	2.928 (2.767–3.098)	< 0.001
Unknown	4.935 (4.618–5.272)	< 0.001	2.353 (2.198–2.520)	< 0.001
Tumor size (mm)				
0–10	0.077 (0.072–0.083)	< 0.001	0.185 (0.164–0.208)	< 0.001
11–20	0.184 (0.173–0.197)	< 0.001	0.324 (0.290–0.361)	< 0.001
21–30	0.399 (0.375–0.426)	< 0.001	0.545 (0.489–0.607)	< 0.001
31–40	0.614 (0.574–0.656)	< 0.001	0.755 (0.675–0.845)	< 0.001
41–50	0.760 (0.708–0.816)	< 0.001	0.845 (0.750–0.953)	0.006
51–60	0.867 (0.803–0.936)	< 0.001	0.911 (0.801–1.038)	0.161
61–70	Reference	–	Reference	–
71–80	1.108 (1.010–1.216)	0.030	1.160 (0.994–1.354)	0.059
>80	1.289 (1.187–1.399)	< 0.001	1.079 (0.931–1.249)	0.313
Regional nodes				
Negative	Reference	–	Reference	–
Positive	3.626 (3.549–3.705)	< 0.001	2.478 (2.422–2.536)	< 0.001
Unknown	3.181 (2.988–3.385)	< 0.001	2.100 (1.970–2.237)	< 0.001
Radiation				
Yes	Reference	–	Reference	–
No	1.437 (1.407–1.467)	< 0.001	1.176 (1.150–1.201)	< 0.001
Unknown	1.635 (1.545–1.730)	< 0.001	1.190 (1.124–1.259)	< 0.001
Size × Hormone^c^	–	–		< 0.001

Abbreviations: CI, confidence interval; ER, estrogen receptor; HR, hazard ratio; PR, progesterone receptor; UD, undifferentiated

a Including American Indian/Alaskan native and Asian/Pacific Islander

b Including divorced, separated, single (never married), and widowed

c We defined an interaction term (size × nodes) to determine whether there was significant interaction between tumor size and hormone receptor status in predicting breast cancer-specific mortality.

### Interaction between tumor size and HoR status regarding BCSM

**Figure 2:**
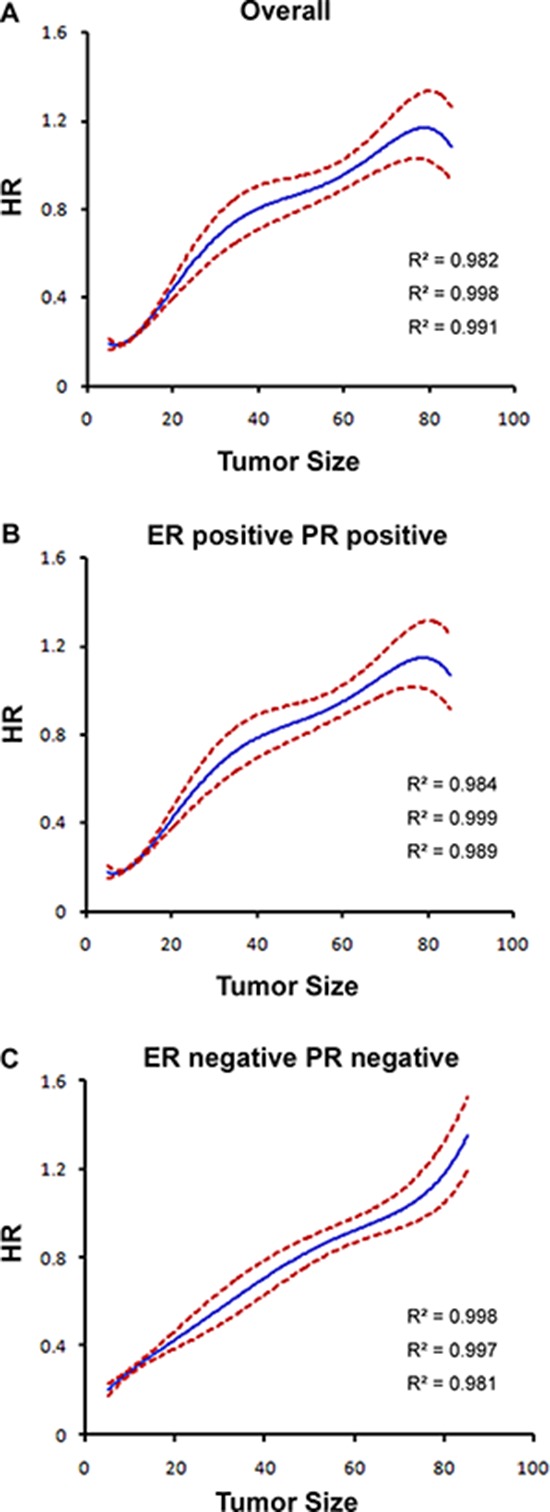
Estimates of hazard ratios (HRs) of breast cancer-specific mortality based on tumor size for different ER/PR status groups using quantic polynomial regression R-squared (R^2^) values are reported. The solid blue lines represent HR estimates, whereas the dashed red lines represent 95% confidence intervals. **A.** The entire cohort. **B.** ER-positive and PR-positive patients. **C.** ER-negative and PR-negative patients.

There was a significant interaction between tumor size and HoR status in determining BCSM in the multivariate analysis (*P* < 0.001; Table 2). The relationship between continuous tumor size and BCSS stratified by ER/PR status was illustrated by a pairwise comparison (Table 3) that revealed differing patterns in the prognostic value of tumor size. In the ER+PR+ group, the HRs for BCSM increased with increasing tumor size until a threshold was reached (approximately 50 mm) (0–10 mm, HR 0.176, 95% CI 0.156–0.198, *P* < 0.001; 11–20 mm, HR 0.308, 95% CI 0.276–0.343, *P* < 0.001; 21–30 mm, HR 0.525, 95% CI 0.471–0.585, *P* < 0.001; 31–40 mm, HR 0.739, 95% CI 0.660–0.827, *P* < 0.001; 41–50 mm, HR 0.831, 95% CI 0.737–0.936, *P* = 0.002). Thereafter, increasing tumor size was no longer related to increased BCSM (51–60 mm, HR 0.904, 95% CI 0.794–1.029, *P* = 0.127; 71–80 mm, HR 1.139, 95% CI 0.976–1.329, *P* = 0.099; > 80 mm, HR 1.070, 95% CI 0.923–1.239, *P* = 0.370). In the entire cohort, the HRs of BCSM were plotted against the different tumor size groups (Figure 2A). The graphed HRs for the ER+PR+ subgroup formed a gradually rising curve that plateaued at the maximum value (Table 3; HR estimates, R-squared (R^2^) values = 0.999; 95% CI, R^2^ values = 0.984 and 0.989; Figure 2B). Similar patterns were observed for the ER+PR− and ER−PR+ subgroups (Table 3; Figure S2A–S2B), with the > 80 mm tumor groups exhibiting borderline significance (ER+PR−, HR 1.253, 95% CI 1.009–1.555, *P* = 0.041; ER−PR+, HR 1.590, 95% CI 1.012–2.498, *P* = 0.044). The HRs for the patients with ER+PR− or ER−PR+ tumors had wide 95% CIs because of the relatively small sample size. The above results suggested that larger tumors (>51 mm) with ER+ and/or PR+ phenotypes potentially represent a unique tumor subtype with an invariable prognosis.

**Table 3:** Pairwise comparisons between different combinations of size and hormone receptor statuses relative to breast cancer-specific mortality^a^

Variable	Hormone receptor status							
	ER+PR+		ER+PR−		ER−PR+		ER−PR−	
	HR (95% CI)	*P*	HR (95% CI)	*P*	HR (95% CI)	*P*	HR (95% CI)	*P*
Tumor size (mm)								
0–10	0.176 (0.156–0.198)	< 0.001	0.144 (0.118–0.175)	< 0.001	0.191 (0.127–0.290)	< 0.001	0.205 (0.183–0.229)	< 0.001
11–20	0.308 (0.276–0.343)	< 0.001	0.285 (0.239–0.340)	< 0.001	0.339 (0.235–0.490)	< 0.001	0.349 (0.317–0.384)	< 0.001
21–30	0.525 (0.471–0.585)	< 0.001	0.461 (0.388–0.548)	< 0.001	0.575 (0.399–0.828)	0.003	0.514 (0.468–0.565)	< 0.001
31–40	0.739 (0.660–0.827)	< 0.001	0.622 (0.520–0.745)	< 0.001	0.690 (0.473–1.008)	0.055	0.637 (0.577–0.702)	< 0.001
41–50	0.831 (0.737–0.936)	0.002	0.713 (0.588–0.863)	0.001	0.888 (0.593–1.330)	0.564	0.777 (0.701–0.862)	< 0.001
51–60	0.904(0.794–1.029)	0.127	0.769 (0.625–0.946)	0.013	0.931 (0.610–1.421)	0.740	0.856 (0.767–0.956)	0.006
61–70	Reference	–	Reference	–	Reference	–	Reference	–
71–80	1.139 (0.976–1.329)	0.099	0.828 (0.639–1.072)	0.152	0.898 (0.541–1.491)	0.678	1.060 (0.927–1.213)	0.392
>80	1.070 (0.923–1.239)	0.370	1.253 (1.009–1.555)	0.041	1.590 (1.012–2.498)	0.044	1.356(1.207–1.523)	< 0.001

Abbreviations: CI, confidence interval; ER, estrogen receptor; HR, hazard ratio; PR, progesterone receptor

a The results of the different combinations of size (rows) and hormone receptor status (columns) are presented at the intersections of the rows and columns. All the results were adjusted using Cox proportional hazards models for year of diagnosis, race, marital status, age, laterality, grade, node, and radiation history.

However, in the ER−PR− group (Table 3), the HRs for BCSM gradually increased with increasing tumor size (0–10 mm, HR 0.205, 95% CI 0.183–0.229, *P* < 0.001; 11–20 mm, HR 0.349, 95% CI 0.317–0.384, *P* < 0.001; 21–30 mm, HR 0.514, 95% CI 0.468–0.565, *P* < 0.001; 31–40 mm, HR 0.637, 95% CI 0.577–0.702, *P* < 0.001; 41–50 mm, HR 0.777, 95% CI 0.701–0.862, *P* < 0.001; 51–60 mm, HR 0.856, 95% CI 0.767–0.956, *P* = 0.006; 71–80 mm, HR 1.060, 95% CI 0.927–1.213, *P* = 0.392; > 80 mm, HR 1.356, 95% CI 1.207–1.523, *P* < 0.001). For this group, the HR was highest in the group with tumors larger than 80 mm. The plotted HRs for this subgroup exhibited a constant increase with increasing tumor size (Table 3; Figure 2C).

To clarify whether the interaction between tumor size and ER/PR status is affected by potential confounders, we performed BCSM analyses using different interaction terms after stratifying by LN status. A significant interaction between tumor size and ERPR status was identified in both LN-negative and LN-positive patients (Table S1). Except for certain small changes, the HR trends in all the luminal subgroups after stratification by LN status were similar to those in the previous analyses (Table S1).

## DISCUSSION

A more comprehensive characterization of this interaction could increase our understanding of breast cancer biology and individualized treatments. Thus, we sought to determine whether there is a significant interaction between tumor size and HoR status in predicting BCSM in subdivided categories, especially in groups with larger tumors. In addition, we hypothesized that for ER+ and/or PR+ tumors, large primary lesions (defined as > 50 mm) may indicate biologically indolent disease and may thus predict different BCSS patterns compared to ER−PR− tumors of a similar size. After adjusting for known breast cancer prognostic factors and accounting for multiple comparisons, we observed a linear effect of increasing size on BCSM in the ER−PR− subsets, a result that is consistent with traditional perspectives [2, 3, 4]. Interestingly, the effect of tumor size on BCSM within the ER+PR+ subgroup was piecewise. Patients with 51–60 mm, 71–80 mm and > 80 mm tumors had a similar BCSM as those with 61–70 mm tumors. These data suggested that increasing tumor size potentially lost its prognostic value in the ER+PR+ group above the threshold of 51 mm. Similar patterns in the prognostic value of tumor size were observed in the ER+PR− and ER−PR+ subgroups.

The following are possible explanations for this interrupted relationship between tumor size and survival. First, luminal breast cancer is a highly heterogeneous disease. For the study cohort herein, luminal tumors refer to ER+PR+, ER+PR− and ER−PR+ breast cancer. Previous studies using microarray technology have indicated that tumor heterogeneity is also present at the gene expression level, and two main luminal breast cancer subtypes have been identified [17, 18, 19]. These subtypes are referred to as luminal A and luminal B, and they have different gene expression profiles, prognoses and treatment responses [17, 19, 20]. A tumor gene signature represents the average of all cells sampled within the tumor. If an aggressive cellular component of a tumor represents a smaller proportion of the total cell population in a larger tumor, its gene signature may be diluted by the bulk of the less aggressive tumor cells. Thus, patients with larger luminal tumors may have similar survival outcomes as patients with smaller tumors.

Second, it is universally accepted that a cancer spreads when metastatic ability is obtained through the accumulation of mutations as the tumor grows to a large size [21, 22]. Moreover, several newly identified cancer genes in ER+ breast cancer have loss-of-function mutations [23, 24]. Certain studies have provided examples to illustrate that mutations can be associated with good prognosis in patients with ER+ breast cancer, e.g., some PIK3CA mutations [25, 26]. It is therefore conceivable that a larger luminal tumor that has accumulated mutations may not confer a worse prognosis.

Third, patients with larger tumors are more likely to receive more advanced treatment regimens compared with patients with smaller luminal tumors; these advanced therapies include more radical surgery, more intensive radiation therapy, more aggressive chemotherapy and extended endocrine therapy. Therefore, patients with tumors larger than 50 mm may not have worse BCSS.

Moreover, the observed relationships essentially remained in all luminal tumors after LN stratification. This finding further supports the idea that the use of molecular profiling in conjunction with tumor size and node status may improve prognostic power.

Our findings have potential implications in both clinical practice and breast cancer research. Larger luminal tumors have a seemingly indolent nature, which may be due to more aggressive treatment and/or intrinsic factors. Because such larger ER+ and/or PR+ tumors most likely have no higher chance of distant dissemination, local-regional treatments might be more crucial. The extension of surgery, systemic therapy options, and timing of adjuvant chemotherapy (before or after radiotherapy) should be individualized. Even after metastasis, larger ER+ and/or PR+ tumors might present no greater possibility of leading to rapid progression or visceral crisis, and thus endocrine therapy or other less aggressive regimens could be taken into consideration for these patients to avoid unnecessary treatment and exposure to side effects. Overall, achieving similar favorable outcomes remains possible in this subgroup of patients. In addition, the findings of this study support a growing body of literature that addresses the importance of molecular subtype classification in addition to LN metastasis and tumor size for predicting survival. In addition to ER/PR profiles, more gene expression profiles are beginning to emerge, and these must be explored to better define tumor signatures [27–32].

Our study has some limitations. First, the SEER database does not contain information regarding HER2/neu status and systemic therapy. Therefore, these potential confounding factors could not be adjusted in our analyses. Second, breast cancer can have a long natural history; thus, a median follow-up of 68 months may not reveal long-term survival differences. Third, the retrospective nature of our study may have introduced bias into the analysis. Despite these limitations, this study is convincing because it is based on a large population and multiple centers.

In conclusion, our study revealed that BCSM does not increase with increasing tumor size in patients with luminal breast cancer lesions greater than 50 mm in size, regardless of LN status. It might be possible to achieve a favorable clinical outcome for patients with this subtype of breast cancer, suggesting that these patients require more individualized treatment. In addition, the biological behavior of this heterogeneous disease warrants further investigation.

## METHODS

### Patients

Data were obtained from the current SEER database, which consists of 18 population-based cancer registries. SEER data are an open access resource for cancer-based epidemiology and survival analyses. SEER*Stat software from the National Cancer Institute (Surveillance Research Program, National Cancer Institute SEER*Stat software, http://www.seer.cancer.gov/seerstat) (Version 8.1.5) was used to identify eligible patients.

The following inclusion criteria were utilized for patient selection: female, pathological diagnosis of invasive ductal carcinoma, unilateral breast cancer, known tumor size, breast cancer as the first and only cancer diagnosis, diagnosis not obtained from a death certificate or autopsy, only one primary site, surgical treatment with either mastectomy or breast-conserving surgery, known ER and PR status, American Joint Committee on Cancer (AJCC) stages I-III, known age at diagnosis, and known time of diagnosis from 1990 to 2010. Pathologic diagnosis was based on the primary site using the International Classification of Disease for Oncology, Third Edition (ICD-O-3). The morphology code for infiltrating duct carcinoma was 8500. Patients diagnosed with breast cancer before 1990 were excluded due to unavailable ER and PR data; patients diagnosed with breast cancer after 2010 were excluded to ensure an adequate follow-up time. Treatment status regarding surgery and irradiation therapy was also obtained for the selected patients. Data regarding chemotherapy and endocrine therapy are not included in SEER; hence, these data were not evaluated. HoR status was analyzed by joint ER and PR statuses (ER+PR+, ER+PR−, ER−PR+, and ER−PR−). We used BCSM as the primary study outcome of the SEER data; BCSM was calculated from the date of diagnosis to the date of breast cancer-specific death. Patients who died of non-breast cancer-related causes were censored regarding the date of death.

This study was based on public data released by the SEER database; we obtained permission to access research data files with the reference number 13539-Nov2013. SEER database data do not require informed consent, and our study was conducted with approval from the Ethical Committee Review Board of Fudan University Shanghai Cancer Center.

### Statistical analysis

Study variables are provided in Table 1; these variables were stratified by joint ER/PR expression. Tumor size was treated as a categorical variable to explore the impact of size on BCSM. Tumors larger than 80 mm were combined due to the limited number of cases. The association of ER/PR status with clinicopathologic parameters was analyzed using the chi-squared (χ2) test. We defined an interaction term (size × ERPR) to determine whether there was a significant interaction between tumor size and ER/PR status in predicting BCSM. Pairwise comparisons were performed between different combinations of ER/PR status and tumor size to determine the presence of significant differences in BCSM.

Survival curves were generated by the Kaplan-Meier method, and differences between the curves were analyzed using the log-rank test. Univariate and multivariate Cox regression models were generated to analyze risk factors for BCSM, and adjusted hazard ratios (HRs) with 95% confidence intervals (CIs) were calculated. The nonlinear effect of continuous tumor size on BCSM was assessed using quantic polynomial regression, and R-squared (R^2^) values are reported. All the statistical analyses were performed using SPSS (version 19.0; SPSS Company). Two-sided *P*-values < 0.05 were considered statistically significant.

## SUPPLEMENTARY FIGURES AND TABLE


