# Supplementary material for: Effect of tumor size on breast cancer-specific survival stratified by joint hormone receptor status in a SEER population-based study
Source: Oncotarget. 2015 May 11;6(26):22985–95. doi: 10.18632/oncotarget.3945 (PMC4673215; doi:10.18632/oncotarget.3945)
Supplement: Supplementary file 1 [file oncotarget-06-22985-s001.pdf]

SUPPLEMENTARY FIGURES AND TABLE

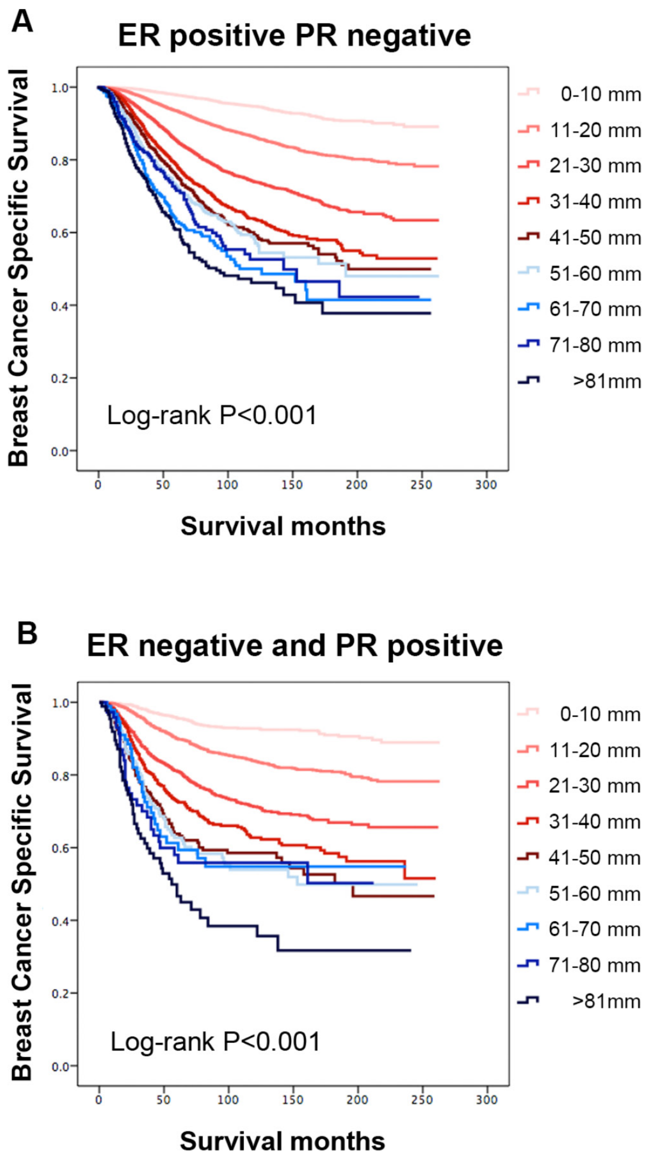

Supplementary Figure S1: Patient survival curves according to tumor size. A. The estrogen receptor (ER)-positive and progesterone receptor (PR)-negative group, $P < 0.001$. **B.** The ER-negative and PR-positive group, $P < 0.001$.

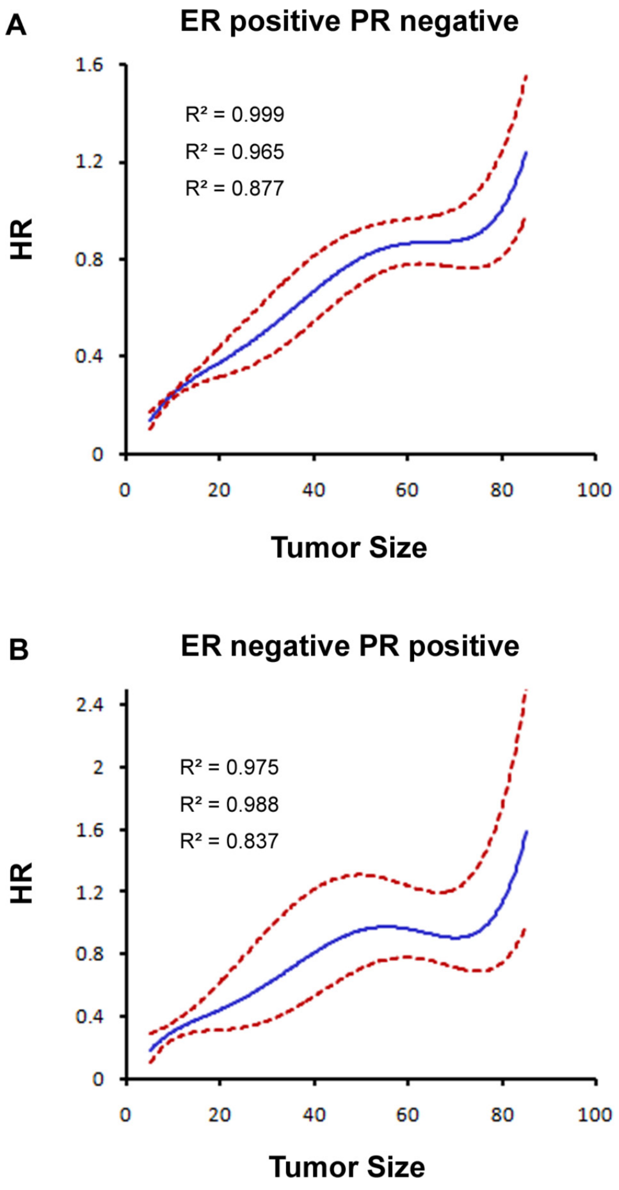

Supplementary Figure S2: Estimates of hazard ratios (HRs) of breast cancer-specific mortality based on tumor size for different ER/PR status groups using quantic polynomial regression. R-squared (R2) values are reported. The solid blue lines represent the HR estimates, whereas the dashed red lines represent the 95% confidence intervals. **A.** ER-positive and PR-negative patients. **B.** ER-negative and PR-positive patients.

Supplementary Table S1: Breast cancer-specific mortality with interaction terms stratified by node status[a]

		Hormone receptor status							
	Tumor	ER+PR+		ER+PR-		ER-PR+		ER-PR-	
Node status	size (mm)	HR (95% CI)	*P*	HR (95% CI)	*P*	HR (95% CI)	*P*	HR (95% CI)	*P*
Positive	0–10	0.194 (0.167–0.226)	< 0.001	0.201 (0.157–0.259)	< 0.001	0.210 (0.126–0.350)	< 0.001	0.234 (0.202–0.272)	< 0.001
	11–20	0.316 (0.280–0.357)	< 0.001	0.296 (0.244–0.359)	< 0.001	0.307 (0.204–0.461)	< 0.001	0.341 (0.306–0.380)	< 0.001
	21–30	0.518 (0.459–0.584)	< 0.001	0.447 (0.370–0.539)	< 0.001	0.555 (0.373–0.827)	0.004	0.475 (0.427–0.527)	< 0.001
	31–40	0.755 (0.667–0.854)	< 0.001	0.625 (0.514–0.759)	< 0.001	0.623 (0.412–0.942)	0.025	0.592 (0.531–0.660)	< 0.001
	41–50	0.840 (0.737–0.958)	0.009	0.720 (0.584–0.886)	0.002	0.763 (0.487–1.196)	0.239	0.724 (0.645–0.813)	< 0.001
	51–60	0.939 (0.815–1.081)	0.381	0.803 (0.644–1.003)	0.053	0.884 (0.557–1.402)	0.599	0.817 (0.723–0.923)	0.001
	61–70	Reference	-	Reference	-	Reference	-	Reference	-
	71–80	1.202 (1.018–1.419)	0.030	0.843 (0.640–1.110)	0.223	0.811 (0.463–1.419)	0.463	1.050 (0.907–1.216)	0.514
	> 80	1.056 (0.898–1.242)	0.510	1.299 (1.029–1.640)	0.028	1.574 (0.969–2.556)	0.067	1.331 (1.172–1.510)	< 0.001
Negative	0–10	0.162 (0.121–0.216)	< 0.001	0.139 (0.080–0.243)	< 0.001	0.256 (0.092–0.716)	0.009	0.251 (0.195–0.323)	< 0.001
	11–20	0.289 (0.217–0.385)	< 0.001	0.303 (0.175–0.525)	< 0.001	0.536 (0.197–1.453)	0.22	0.461 (0.362–0.587)	< 0.001
	21–30	0.512 (0.384–0.683)	< 0.001	0.537 (0.309–0.932)	0.027	0.820 (0.301–2.233)	0.699	0.735 (0.577–0.936)	0.013
	31–40	0.685 (0.509–0.921)	0.012	0.682 (0.388–1.199)	0.184	1.136 (0.407–3.169)	0.807	0.919 (0.717–1.177)	0.503
	41–50	0.751 (0.547–1.030)	0.075	0.784 (0.437–1.407)	0.414	1.624 (0.561–4.697)	0.371	1.145 (0.883–1.485)	0.307
	51–60	0.744 (0.519–1.067)	0.108	0.705 (0.364–1.368)	0.302	1.178 (0.378–3.673)	0.777	1.087 (0.817–1.446)	0.568

(*Continued*)

Node status	Tumor size (mm)	Hormone receptor status							
		ER+PR+		ER+PR-		ER-PR+		ER-PR-	
		HR (95% CI)	*P*	HR (95% CI)	*P*	HR (95% CI)	*P*	HR (95% CI)	*P*
	61–70	Reference	-	Reference	-	Reference	-	Reference	-
	71–80	0.869 (0.545–1.385)	0.554	0.465 (0.166–1.307)	0.146	0.761 (0.169–3.429)	0.722	1.253 (0.872–1.800)	0.222
	> 80	1.198 (0.813–1.765)	0.361	1.167 (0.586–2.322)	0.66	1.159 (0.288–4.660)	0.836	1.444(1.051–1.984)	0.024

Abbreviations: CI, confidence interval; ER, estrogen receptor; HR, hazard ratio; PR, progesterone receptor
[a]All the results were adjusted using Cox proportional hazards models for year of diagnosis, race, marital status, age, laterality, grade, node, and radiation history.
